# Pitfalls and Limitations of the Vesical Imaging-Reporting and Data System (VI-RADS): Challenges in the Multiparametric Magnetic Resonance Imaging (mpMRI) Assessment of Bladder Cancer

**DOI:** 10.7759/cureus.99197

**Published:** 2025-12-14

**Authors:** José Laert, Nuno Lupi Manso, Tomás França de Santana, Pedro O Santos

**Affiliations:** 1 Radiology Department, Hospital CUF Tejo, Lisbon, PRT

**Keywords:** bladder cancer, multiparametric magnetic resonance imaging (mpmri), transurethral resection of bladder tumour (turbt), uro-radiology, vesical imaging-reporting and data system (vi-rads)

## Abstract

The Vesical Imaging-Reporting and Data System (VI-RADS) has emerged as a standardised scoring system for assessing detrusor muscle invasion in patients with urothelial bladder cancer using multiparametric magnetic resonance imaging (mpMRI). Accurate staging is critical for guiding treatment decisions, including bladder-sparing strategies and radical cystectomy.

We performed a comprehensive literature review, complemented by illustrative images from real cases at our institution, to highlight potential pitfalls, limitations, and technical challenges in VI-RADS interpretation. Post-transurethral resection of bladder tumour (post-TURBT) changes, intravesical therapy, and neoadjuvant chemotherapy may mimic residual tumour, affecting both diffusion-weighted imaging and dynamic contrast-enhanced sequences. Specific tumour locations, such as the ureteric orifices, trigone, or bladder neck, as well as flat or small lesions, may complicate accurate staging. Non-urothelial histologies, extravesical tumours, and bladder diverticula can also lead to misinterpretation, while technical factors such as bladder distension, motion artefacts, and slice thickness can impact image quality and scoring accuracy.

VI-RADS remains a valuable tool for the non-invasive staging of bladder cancer, but recognition of its limitations and potential pitfalls is essential to prevent misdiagnosis and inappropriate management. Careful integration of clinical history, cystoscopy, histopathology, and meticulous mpMRI technique improves diagnostic confidence and guides optimal patient care.

## Introduction and background

Bladder cancer (BC) represents a significant public health concern, ranking among the 10 most common malignancies worldwide and carrying the highest lifetime treatment costs per patient [1,2]. The epidemiological profile is characterized by a strong male predominance, with a male-to-female ratio of approximately 4:1, an increasing incidence with advancing age, and the majority of cases and mortality occurring in less developed regions [2,3].

BC is predominantly driven by environmental and stochastic factors, with over 80% of cases attributed to external exposures and approximately 15% unrelated to carcinogen exposure. Smoking is the main risk factor, accounting for around half of all cases and conferring a 2.5-fold increased risk, including long-term low-intensity smokers. Other significant risk factors include occupational and environmental exposures to aromatic amines, aniline dyes, arsenic in drinking water, iatrogenic pelvic radiation, and long-term use of drugs such as cyclophosphamide and certain analgesics. Chronic bladder irritation or infection, congenital anomalies (e.g., urachal remnants, exstrophy), and genetic syndromes such as Lynch or Cowden also contribute to the aetiology of the disease [1,2,4,5].

More than 90% of BC cases are urothelial carcinomas [4,6], classified into low-grade and high-grade, the latter subdivided into forms with or without muscular invasion [7]. Non-muscle-invasive bladder cancer (NMIBC), which represents 70-85% of initial diagnoses [8], includes stages Ta (papillary), T1 (lamina propria invasion), and carcinoma in situ (CIS). Its primary therapeutic goal is to prevent recurrence and progression while preserving quality of life. Muscle-invasive bladder cancer (MIBC), which is more aggressive and accounts for 15-30% of initial cases, comprises stages T2 to T4 [6,7].

Assessment of detrusor muscle invasion is fundamental for the staging, management, and prognosis of BC. Traditionally, diagnosis and staging rely on cystoscopy and transurethral resection of bladder tumour (TURBT), which is considered the gold standard. TURBT enables the resection of all visible lesions for histopathological diagnosis and staging, with the inclusion of detrusor muscle in the specimen being essential [4,6,9]. However, one of its main limitations is the potential underestimation of muscle invasion. Even when the detrusor muscle is included in the initial specimen, TURBT often fails to achieve complete tumour removal, leading to residual disease, early recurrence, and incorrect treatment allocation [10,11]. Additional limitations include its invasive nature, the risk of complications such as pain, haemorrhage, and urinary tract infections [6], and interobserver variability among pathologists in assessing muscular invasion [4,10]. These intrinsic challenges highlight the need for more accurate, non-invasive staging tools to improve preoperative assessment and guide appropriate management.

Multiparametric magnetic resonance imaging (mpMRI) of the bladder has emerged as a superior tool for T-staging of BC, overcoming many of the limitations of TURBT and computed tomography (CT). mpMRI combines anatomical sequences (T2-weighted imaging (T2-WI)) with functional sequences such as diffusion-weighted imaging (DWI) and dynamic contrast-enhanced (DCE) imaging, which improve tumour detection and differentiation between superficial and invasive lesions [9,12].

To standardise the acquisition and interpretation of bladder MRI, the Vesical Imaging-Reporting and Data System (VI-RADS) was developed in 2018 [7]. VI-RADS is a five-point scoring system that evaluates the probability of muscular invasion, playing a crucial role in accurate disease staging.

Validation studies have shown that mpMRI with VI-RADS scoring has a pooled sensitivity of 90-92% and a specificity of 84-86% for distinguishing NMIBC from MIBC, using a VI-RADS cut-off score of ≥3 [4]. Accurate staging is vital, as prognosis and management of BC depend on tumour stage and grade [1,13]. mpMRI, particularly when integrated with VI-RADS, enables more refined risk stratification, guiding treatment decisions such as the need for neoadjuvant chemotherapy (NAC) before radical cystectomy or eligibility for bladder-sparing therapies [7,14].

This review highlights the main pitfalls, limitations, and technical challenges in VI-RADS interpretation, with a focus on clinical, anatomical, histological, and post-treatment factors that may affect staging accuracy. A literature search was conducted on PubMed for articles published over the past decade using the terms "bladder cancer", "VI-RADS", and "mpMRI". The discussion is supported by representative imaging cases from our institution, all obtained with informed patient consent for publication.

## Review

Components of mpMRI and VI-RADS classification

The primary aim of VI-RADS is to determine the probability of detrusor muscle invasion in patients with confirmed urothelial neoplasia. Differentiating NMIBC from MIBC is essential for treatment decisions, emphasising the need for accurate assessment of muscle infiltration before therapy [7]. VI-RADS was initially designed for untreated patients or those who had undergone only diagnostic TURBT, although its application is now more flexible [2,4].

Bladder mpMRI integrates anatomical and functional sequences. The key sequences are T2-WI, DWI, and DCE imaging [7,15]. VI-RADS uses a five-point scale to assess the likelihood of muscular invasion, combining evaluations from each sequence into a global score [7].

T2-WI (Structural Category)

These sequences should be the first considered for VI-RADS scoring, given their high spatial resolution in assessing the integrity of the muscularis propria. The muscle appears hypointense on T2-WI, and interruption of this low-signal line may suggest invasion [4,9,16].

DWI (Diffusion-Weighted Category)

This is crucial for detecting and staging muscular invasion. The tumour appears hyperintense on DWI with corresponding hypointensity on apparent diffusion coefficient (ADC) maps. DWI is regarded as the dominant sequence for risk estimation, particularly when image quality is optimal, followed by DCE imaging [4,9,16].

DCE Imaging (Contrast-Enhanced Category)

This improves detection and differentiation between superficial and invasive lesions, showing the early enhancement of the inner layer and tumour. DCE imaging is considered the second dominant sequence, especially when DWI quality is suboptimal [4,9,16].

The inner layer (urothelium and lamina propria) normally enhances early on DCE imaging and is not visible on T2-WI or DWI. However, in cases of oedema or inflammation, it may appear as a hyperintense structure on T2-WI and hypointense on DWI [4,7].

The VI-RADS scale is based on a five-point classification applied individually to the key imaging sequences (T2-WI, DWI, and DCE imaging). The overall score is derived from the combination of these evaluations, resulting in five categories that express the probability of detrusor muscle invasion. Categories 1 and 2 correspond to tumours with low probability of muscular involvement, while categories 4 and 5 indicate high suspicion of invasion. Category 3 is considered equivocal for muscular invasion [5].

Pitfalls and limitations of VI-RADS

Equivocal Cases (VI-RADS 3)

**Figure 1:**
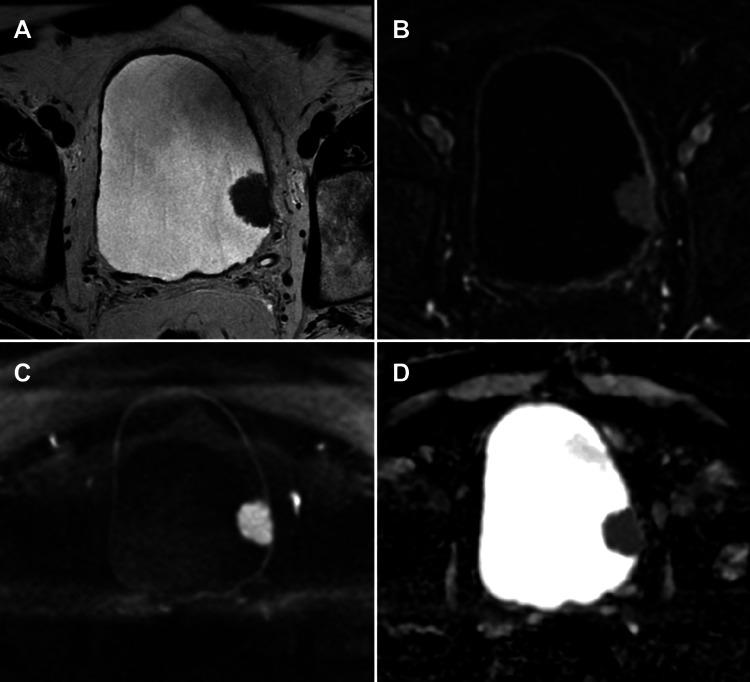
mpMRI pitfall: VI-RADS 3 in a non-muscle-invasive exophytic bladder tumour (A) Axial T2-WI, (B) axial DCE imaging, (C) axial DWI, and (D) axial ADC map. mpMRI showing a tumour on the left lateral bladder wall classified as VI-RADS 3 due to equivocal muscular invasion. The tumour is exophytic without a stalk, and there is no observation of a thickened inner layer with high T2 signal, which would classify it as VI-RADS 2. Histopathology following TURBT confirmed a non-muscle-invasive tumour. The patient is under follow-up with no evidence of tumour recurrence. This case illustrates a potential pitfall where imaging may overestimate muscle invasiveness in exophytic tumours lacking typical inner layer changes. Patient consent for publication was obtained. mpMRI: multiparametric magnetic resonance imaging; VI-RADS: Vesical Imaging-Reporting and Data System; T2-WI: T2-weighted imaging; DCE: dynamic contrast-enhanced; DWI: diffusion-weighted imaging; ADC: apparent diffusion coefficient; TURBT: transurethral resection of bladder tumour

VI-RADS category 3 is recognised as the "equivocal" category, representing the point at which mpMRI cannot reliably determine the risk of bladder muscle invasion. This ambiguity is critical, as accurate staging is essential for guiding prognosis and management, distinguishing NMIBC from MIBC [3,5]. On imaging, Structural Category 3 (T2-WI), Contrast Category 3 (DCE imaging), and Diffusion Category 3 (DWI) are characterised by the absence of category 2 findings but without the clear interruption of the low-signal-intensity muscularis propria [2]. This includes exophytic tumours without a stalk or sessile/broad-based tumours without a clearly thickened inner layer of high signal on T2-WI but also without obvious interruption of the muscularis propria [4,5,9] (Figure 1).

When a tumour is classified as VI-RADS 3, surgeons should be alerted that the degree of invasion is difficult to assess, requiring careful consideration of treatment options [9,14]. For VI-RADS 3 lesions, if the initial TURBT indicates NMIBC, a second TURBT or complete resection may be required for accurate pathological staging [17].

Post-procedural Oedema and Inflammation

TURBT, bladder biopsies, and intravesical treatments such as instillation of chemotherapeutic agents (e.g., Bacillus Calmette-Guérin (BCG)) or NAC can induce oedema and inflammation of the bladder wall and perivesical fat [9]. In the long term, TURBT may cause fibrosis and chronic inflammation, which replace the normal bladder wall components and often result in wall thickening [7]. NAC can compromise the reliability of restaging TURBT, resulting in pathological understaging in 32% of patients compared with radical cystectomy specimens [14].

**Figure 2:**
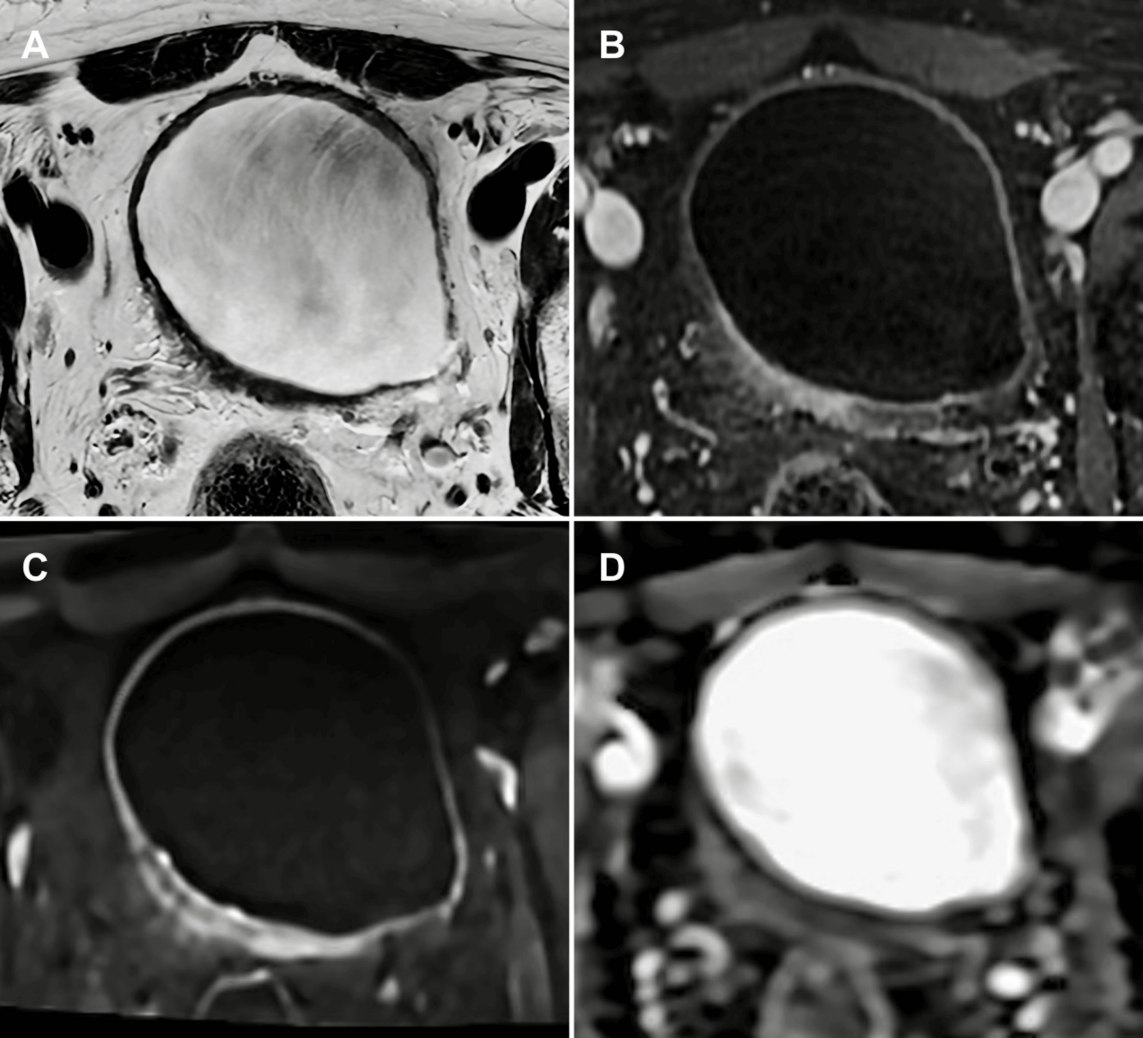
mpMRI of post-TURBT bladder wall changes: differentiating inflammation from residual tumour (A) Axial T2-WI, (B) axial DCE imaging, (C) axial DWI, and (D) axial ADC map. mpMRI of a patient who underwent TURBT for a tumour located on the right posterior wall of the bladder, performed two weeks prior. There is segmental thickening of the bladder wall at the procedural site, with segmental enhancement on the dynamic contrast-enhanced study, indicative of post-procedural inflammatory changes. These inflammatory alterations may overlap with a potential residual tumour, complicating differentiation. The DWI sequence shows high signal at the site, but the ADC map does not demonstrate corresponding low signal to suggest residual tumour, favouring inflammation rather than neoplasia. The absence of significant diffusion restriction aids in distinguishing inflammatory changes from residual tumour, emphasising the importance of multiparametric interpretation using VI-RADS in the post-TURBT setting. Patient consent for publication was obtained. mpMRI: multiparametric magnetic resonance imaging; VI-RADS: Vesical Imaging-Reporting and Data System; T2-WI: T2-weighted imaging; DCE: dynamic contrast-enhanced; DWI: diffusion-weighted imaging; ADC: apparent diffusion coefficient; TURBT: transurethral resection of bladder tumour

Post-treatment inflammation and oedema may cause bladder wall hyperintensity on T2-WI, complicating the distinction between residual tumour and benign treatment-related changes, lowering the sensitivity, specificity, and accuracy of T2-WI in predicting complete pathological response after chemoradiotherapy [7,9]. Post-procedural inflammatory changes may show diffuse mural enhancement on DCE imaging, which can overlap with tumour enhancement and hinder differentiation [4,7,9] (Figure 2).

**Figure 3:**
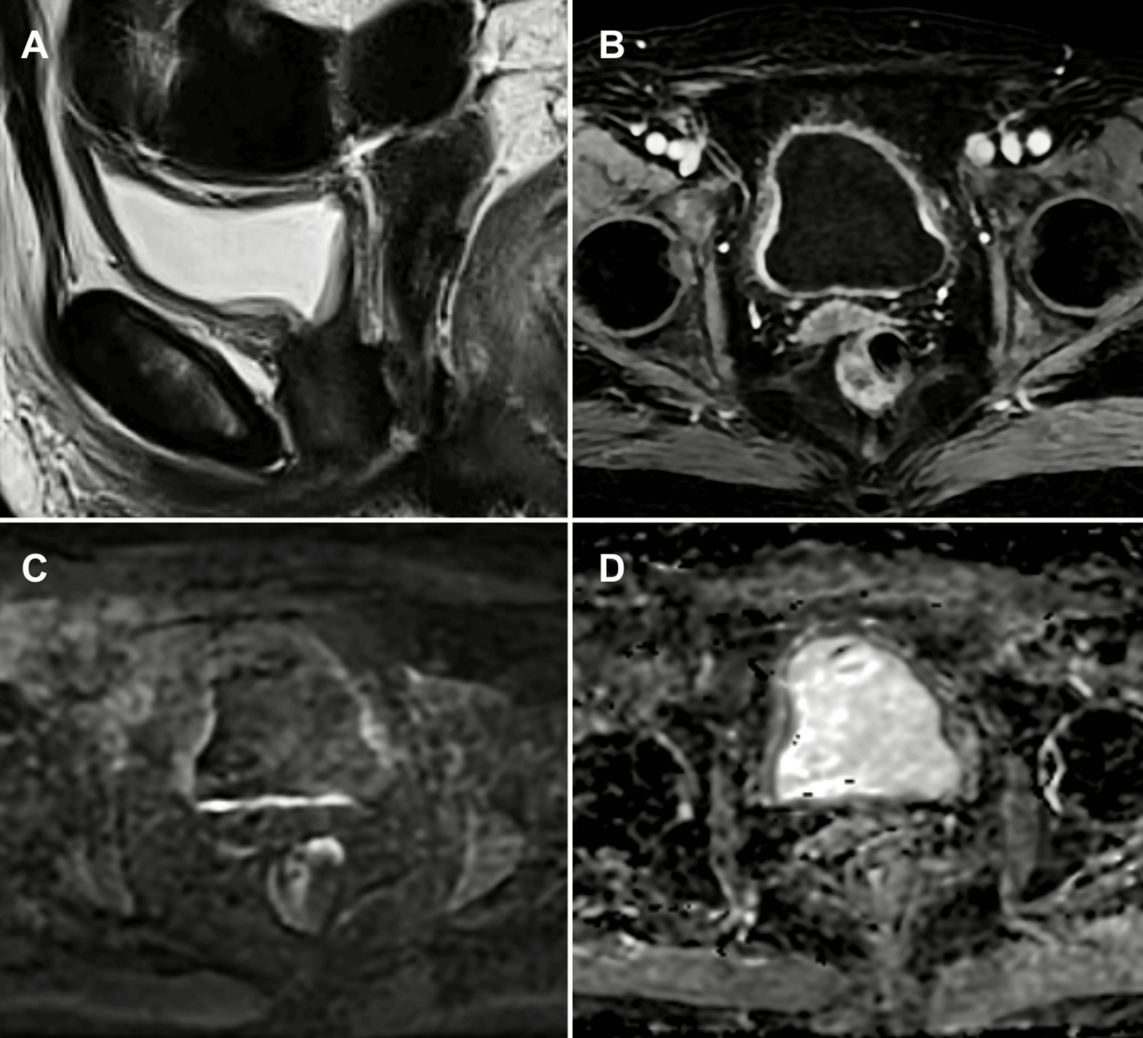
mpMRI of post-BCG bladder wall changes: distinguishing reactive inflammation from residual tumour (A) Sagittal T2-WI, (B) axial DCE imaging, (C) axial DWI, and (D) axial ADC map. mpMRI of a bladder tumour patient treated with TURBT followed by intravesical BCG instillation. The T2-weighted image demonstrates mucosal thickening resembling bullous oedema of the bladder wall. The DCE image shows diffuse enhancement of the bladder wall. Despite diffuse hyperintensity within the bladder wall on DWI and corresponding hypointensity on the ADC map, these features are interpreted as reactive changes related to recent treatment rather than tumour recurrence or residual disease. This case exemplifies the challenge of differentiating post-treatment inflammatory changes from viable tumour using mpMRI. Patient consent for publication was obtained. mpMRI: multiparametric magnetic resonance imaging; T2-WI: T2-weighted imaging; DCE: dynamic contrast-enhanced; DWI: diffusion-weighted imaging; ADC: apparent diffusion coefficient; TURBT: transurethral resection of bladder tumour; BCG: Bacillus Calmette-Guérin

Chronic fibrosis and inflammatory tissue have lower cellular density than cancer, so diffusion restriction is less pronounced. However, inflammatory processes such as cystitis, urothelial papilloma, and post-TURBT changes may display high signal on DWI and ADC maps, mimicking tumour due to oedematous or fibrotic tissue. Substantial overlap of ADC values can occur between recurrent tumours and therapy-related inflammatory reactions [12] (Figure 3). DWI is considered superior to DCE imaging in differentiating recurrent tumour from post-surgical inflammation or fibrosis [7,18].

VI-RADS was initially designed for untreated patients or those who had undergone only diagnostic TURBT [4,7]. Its application is not recommended in patients with repeated TURBT or other treatments, although use has become more flexible. Tumours scored VI-RADS 4 and 5 may have high false-positive rates due to NAC-induced inflammatory response, potentially leading to overstaging [14].

It is essential that mpMRI be performed before TURBT or at least 2-8 weeks after TURBT, bladder biopsy, or intravesical treatment [4,7,9]. Some studies suggest a shorter interval of 1-2 weeks between bladder procedures and MRI to avoid the overestimation of extravesical spread due to post-biopsy inflammatory changes [18].

Tumour Anatomical Location

**Figure 4:**
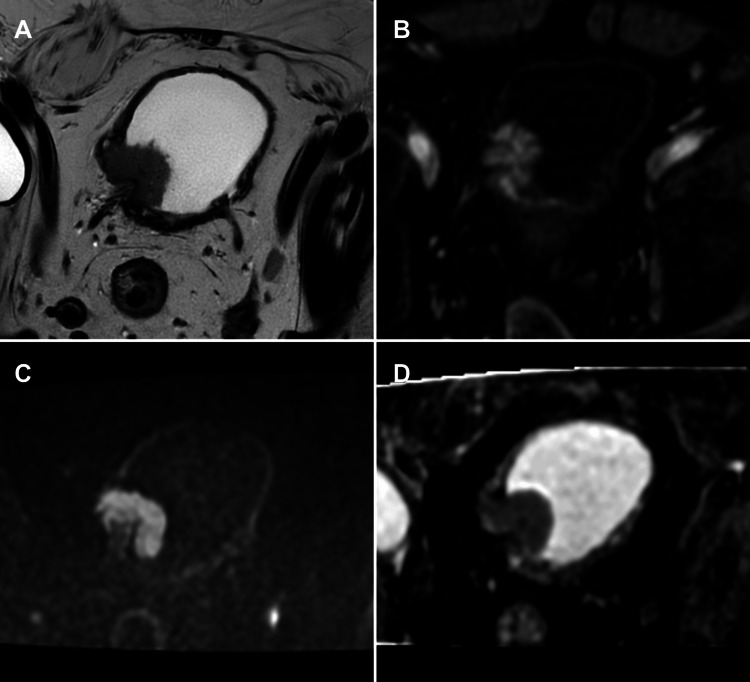
mpMRI of bladder tumour at the ureterovesical junction: limitations in assessing muscular invasion (A) Axial T2-WI, (B) axial DCE imaging, (C) axial DWI, and (D) axial ADC map. mpMRI showing a tumour at the left ureterovesical junction with limited assessment of muscular invasion due to the curved morphology in this region, which impairs visualisation of the tumour's maximum depth of invasion. There is no uretero-hydronephrosis as well as no tumour detected within the ureteral lumen. VI-RADS 4 was assigned based on imaging features, and histopathological evaluation confirmed muscular invasion. This case underscores the challenges in accurately staging tumour invasion at anatomically complex sites such as the ureterovesical junction, highlighting a key pitfall in imaging interpretation. Patient consent for publication was obtained. mpMRI: multiparametric magnetic resonance imaging; VI-RADS: Vesical Imaging-Reporting and Data System; T2-WI: T2-weighted imaging; DCE: dynamic contrast-enhanced; DWI: diffusion-weighted imaging; ADC: apparent diffusion coefficient

**Figure 5:**
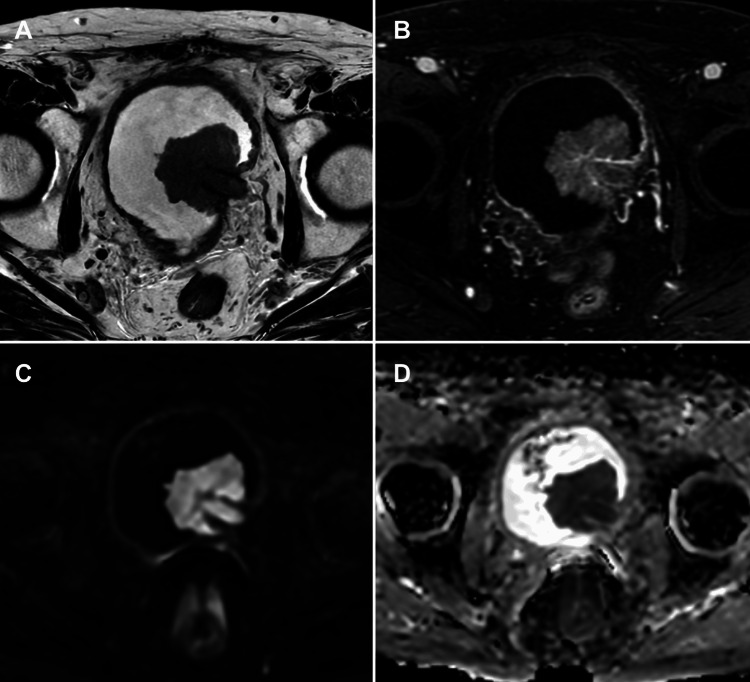
Pitfall in assessing muscular invasion near the ureterovesical junction on mpMRI (A) Axial T2-WI, (B) axial DCE imaging, (C) axial DWI, and (D) axial ADC map. Case of a tumour located at the left ureteric orifice, associated with uretero-hydronephrosis (not shown). There is low signal intensity between the tumour and the bladder wall at the ureterovesical junction on TWI and DWI sequences, which may lead to the underestimation of the degree of muscular invasion. Based on these imaging findings and the presence of tumour within the distal ureter, a VI-RADS 5 classification was assigned. Histopathological analysis of the surgical specimen confirmed muscular invasion and involvement of the ureter. This case highlights a known pitfall in imaging assessment near the ureterovesical junction, where anatomic complexity and signal characteristics can obscure the true extent of tumour. Patient consent for publication was obtained. mpMRI: multiparametric magnetic resonance imaging; VI-RADS: Vesical Imaging-Reporting and Data System; T2-WI: T2-weighted imaging; DCE: dynamic contrast-enhanced; DWI: diffusion-weighted imaging; ADC: apparent diffusion coefficient

Tumours located in the trigone, bladder neck, and ureteric orifices are particularly challenging to stage due to the limited visibility of the maximal depth of tumour invasion. In these curved areas, assessment of muscular invasion can be difficult, with high interobserver variability (Figure 4). One study reported the underestimation of muscular invasion in MIBC located over the ureteric orifice, particularly in cases with hydronephrosis [15]. In tumours adjacent to the ureteric orifice associated with uretero-hydronephrosis, low-signal intensity on DWI between the tumour and the bladder wall may be misinterpreted as preserved muscle, potentially leading to the false-negative assessment of muscular invasion (Figure 5). In such cases, on DCE imaging, 3D acquisition is preferred for its higher spatial resolution, which may aid in delineating tumours in these anatomically curved locations [12].

Additionally, obtaining adequate muscular sampling during biopsy or TURBT in these regions may be challenging, further complicating the histopathological confirmation of muscular invasion [4,15].

Small and Flat Tumours

Small tumours (<1 cm) may be difficult to detect on DWI due to limited spatial resolution and insufficient contrast with the normal bladder wall. Scattered infiltrative tumours may also fail to generate enough signal on DWI to be distinguished from the normal bladder wall, leading to understaging [7,9]. The low spatial resolution and poor signal-to-noise ratio of DWI limit its applicability in the assessment of small tumours or a thin bladder wall with overdistension, making it susceptible to numerous artefacts [16].

**Figure 6:**
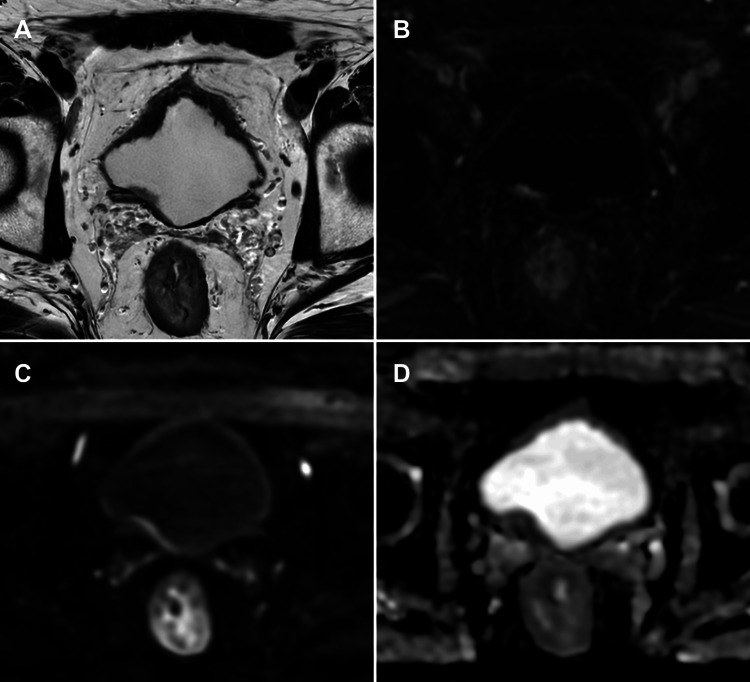
Flat bladder tumour at the ureterovesical junction: challenges in evaluating muscular invasion on mpMRI (A) Axial T2-WI, (B) axial DCE imaging, (C) axial DWI, and (D) axial ADC map. mpMRI showing a flat, elongated tumour on the right posterior bladder wall at the ureterovesical junction. The flat morphology complicates the evaluation of muscular invasion, as laterally spreading lesions within the inner bladder layer cause minimal distortion or thickening. Anatomical complexity at the ureterovesical junction further limits staging accuracy. Based on imaging features, a VI-RADS 2 classification was assigned. Histological analysis of the TURBT specimen confirmed the absence of muscular invasion. This case exemplifies a common limitation of VI-RADS when assessing flat tumours in challenging anatomical locations. Patient consent for publication was obtained. mpMRI: multiparametric magnetic resonance imaging; VI-RADS: Vesical Imaging-Reporting and Data System; T2-WI: T2-weighted imaging; DCE: dynamic contrast-enhanced; DWI: diffusion-weighted imaging; ADC: apparent diffusion coefficient; TURBT: transurethral resection of bladder tumour

Flat lesions that spread laterally within the inner bladder layer, causing minimal wall distortion or thickening, are hard to evaluate (Figure 6). DCE imaging sequences can be particularly valuable for assessing small lesions and are especially helpful for less experienced radiologists to compensate for limitations of DWI [4].

CIS represents a highly aggressive form of flat tumour. Given the limitations of MRI in detecting flat lesions and the infiltrative nature of CIS, photodynamic diagnosis (PDD), which uses hexaminolevulinate to guide resection (PDD-TURBT), has proven to be significantly more sensitive than conventional white-light cystoscopy for detecting malignant bladder lesions, particularly CIS [19].

Non-urothelial and Extravesical Tumours

**Figure 7:**
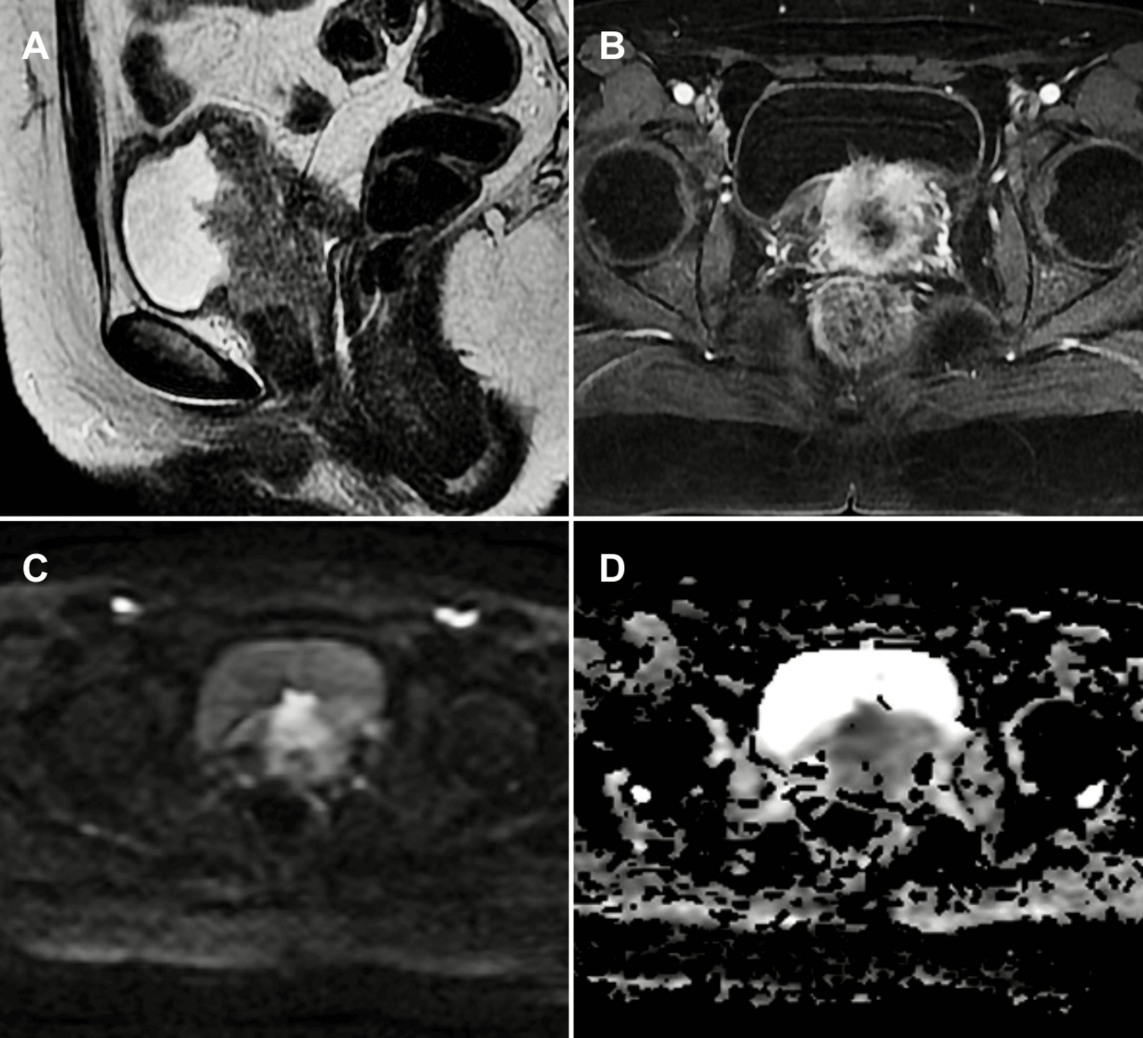
Recurrent cervical cancer with bladder invasion: restricted applicability of VI-RADS to non-urothelial tumours (A) Sagittal T2-WI, (B) axial DCE imaging, (C) axial DWI, and (D) axial ADC map. Case showing a malignant cervical tumour with local recurrence invading the posterior bladder wall, vagina, and urethra. The lesion appears as an extensive tumour mass in the posterior bladder wall, exhibiting heterogeneous enhancement on the intravenous contrast study and diffusion restriction. As VI-RADS was developed specifically for urothelial carcinoma, its applicability to other histological types, particularly metastatic or non-urothelial tumours, is limited. Therefore, correlation with cystoscopy, histopathology, and the patient's clinical history is essential for accurate diagnosis and management. Patient consent for publication was obtained. VI-RADS: Vesical Imaging-Reporting and Data System; T2-WI: T2-weighted imaging; DCE: dynamic contrast-enhanced; DWI: diffusion-weighted imaging; ADC: apparent diffusion coefficient

VI-RADS interpretation can be challenging when the bladder is involved by extravesical tumours or neoplasms with histology other than urothelial carcinoma. In such cases, lesions originating from the uterus, prostate, colon, or rectum may mimic bladder carcinoma and lead to staging errors (Figure 7). Prostate cancer, for example, can directly invade the bladder neck, making it difficult to identify the lesion's epicentre, although clues such as continuity with altered prostatic tissue, presence of sclerotic bone metastases, or elevated serum prostate-specific antigen (PSA) support a prostatic origin [4,9].

Similarly, distant metastatic tumours (stomach, breast, lung) can infiltrate the bladder, causing diffuse bladder wall thickening and complicating mpMRI interpretation [12].

Other primary bladder malignancies beyond urothelial carcinoma, such as squamous cell carcinoma, adenocarcinoma, lymphoma, neuroendocrine neoplasms, mesenchymal tumours, or histological variants of urothelial carcinoma, also constitute pitfalls. These entities may present with non-specific imaging findings overlapping with classic urothelial carcinoma but generally exhibit more aggressive behaviour, inferior therapeutic response, and worse prognosis [7,9,20]. Since VI-RADS was specifically developed for urothelial carcinoma, its applicability to other histologies is limited, making correlation with cystoscopy and histopathology essential.

Bladder Diverticula

Bladder diverticula are a significant pitfall in mpMRI interpretation and VI-RADS application, mainly because they lack a true muscular layer. Most are acquired pseudodiverticula, in which the mucosa and lamina propria herniate through defects in the muscular fibres. The absence of muscularis propria compromises TNM classification, as tumours originating within diverticula cannot be staged as T2, since the depth-of-invasion criterion does not conventionally apply [3,4,13].

Consequently, VI-RADS is inadequate for assessing neoplasms located in bladder diverticula, as it was specifically designed to estimate the probability of detrusor muscle invasion. Furthermore, mpMRI has limitations in determining tumour extent and potential perivesical fat infiltration in these cases. Given these diagnostic and staging challenges, radical cystectomy is often considered the standard treatment to minimise residual tumour and recurrence risk [9].

Benign Bladder Pathologies

The presence of benign bladder abnormalities represents a major challenge in mpMRI interpretation and VI-RADS application, as many of these lesions can mimic urothelial carcinoma, leading to diagnostic errors, overstaging, or unnecessary interventions. Correlation with clinical history, treatment background, and cystoscopic findings is essential to improve diagnostic accuracy and avoid unwarranted radical treatment [4,7].

Among these entities, benign mesenchymal tumours such as leiomyomas typically appear hypointense on T2-WI, appear hypointense on DWI (without diffusion restriction), and have well-defined margins, often covered by normal bladder mucosa, a useful finding for differentiating them from invasive malignancies. Paragangliomas may present as intraluminal polypoid masses with T2-WI hyperintensity and intense contrast enhancement, sometimes accompanied by a "salt-and-pepper" pattern due to areas of vascular flow. Solitary fibrous tumours tend to show diffuse T2-WI hypointensity with progressive delayed enhancement, consistent with their fibrous nature [9,20].

Chronic inflammatory changes, such as cystic and glandular cystitis, usually present with focal or diffuse wall thickening, low signal on T1-WI and T2-WI, and post-contrast enhancement. They can form flat or exophytic lesions, often associated with hypervascular pedicles, but preserve the low-intensity line of muscularis propria on T2-WI, a feature that helps exclude invasion. Inflammatory myofibroblastic tumours (IMTs), although benign, are particularly challenging: they appear heterogeneous on T2-WI, with a hyperintense central area (oedematous or myxoid) surrounded by a hypointense periphery (fibrous or cellular tissue); post-contrast, there is pronounced peripheral enhancement with reduced central uptake, a pattern that may be confused with tumoural necrosis [20].

Other situations include ectopic prostatic tissue, which may appear as a small polypoid lesion near the bladder neck, simulating a primary tumour. Blood clots or luminal debris may also resemble cancer but do not enhance with contrast [4].

Technical factors

Patient Preparation and Bladder Distension

Inadequate bladder distension may hinder the proper visualisation of the bladder wall and detrusor muscle, causing the wall to appear thickened or irregular or limiting the detection of small tumours. Overdistension can result in motion artefacts due to patient discomfort [7,9,18]. In one study, 75% of cases excluded for poor image quality were due to inadequate bladder distension [18]. Optimal preparation involves instructing the patient to void 1-2 hours prior and/or drink 500-1000 ml of water 30 minutes before MRI to achieve optimal distension (~300 ml) [7,9].

Motion and Susceptibility Artefacts

Intestinal peristalsis can cause motion and susceptibility artefacts. Administration of antispasmodic agents (e.g., butylscopolamine bromide or glucagon) can minimise these effects [4,9,21].

Air in the bladder (after cystoscopy or catheterisation) can distort DWI due to susceptibility artefacts. An interval of 2-3 days between these procedures and MRI is recommended [4,7,9].

Slice Thickness

Slice thickness is a critical factor for spatial resolution. For T2-WI sequences, a slice thickness of 3-4 mm is recommended to maximise spatial resolution while maintaining signal-to-noise ratio [4,7]. For DCE imaging, 3D acquisition is preferred for higher spatial resolution [7]. Limited spatial resolution may hinder differentiation between T2b and T3a stages. Bladder tumours smaller than 1 cm may be missed on DWI due to low spatial resolution and insufficient tissue contrast between small tumours and the normal bladder wall [7].

## Conclusions

BC staging requires accurate differentiation between NMIBC and MIBC for proper therapeutic planning and prognosis assessment. TURBT remains the gold standard but has significant limitations, including understaging and sampling error.

Bladder mpMRI with VI-RADS offers a non-invasive, reproducible method for staging, with high diagnostic accuracy. It standardises acquisition, interpretation, and reporting, improving communication between radiologists and urologists.

VI-RADS has become an essential tool in BC imaging, complementing cystoscopy and histology and guiding multidisciplinary decision-making. However, pitfalls remain, including equivocal cases (VI-RADS 3), post-procedural inflammation, anatomical challenges, flat tumours, diverticula, benign mimickers, and technical factors. Radiologists must be aware of these limitations to avoid misinterpretation.

## References

[1] Antoni S, Ferlay J, Soerjomataram I, Znaor A, Jemal A, Bray F (2017). Bladder cancer incidence and mortality: a global overview and recent trends. Eur Urol.

[2] Panebianco V, Pecoraro M, Del Giudice F (2022). VI-RADS for bladder cancer: current applications and future developments. J Magn Reson Imaging.

[3] Caglic I, Panebianco V, Vargas HA (2020). MRI of bladder cancer: local and nodal staging. J Magn Reson Imaging.

[4] Fávero Prietto Dos Santos J, Ghezzi CL, Pedrollo IM (2024). Practical guide to VI-RADS: MRI protocols, lesion characterization, and pitfalls. Radiographics.

[5] Pecoraro M, Takeuchi M, Vargas HA, Muglia VF, Cipollari S, Catalano C, Panebianco V (2020). Overview of VI-RADS in bladder cancer. AJR Am J Roentgenol.

[6] Zhu CZ, Ting HN, Ng KH, Ong TA (2019). A review on the accuracy of bladder cancer detection methods. J Cancer.

[7] Panebianco V, Narumi Y, Altun E (2018). Multiparametric magnetic resonance imaging for bladder cancer: development of VI-RADS (Vesical Imaging-Reporting And Data System). Eur Urol.

[8] Reddy BV, Gali KV, Chawla A, Singh A, Bhaskara SP, Hegde P (2024). Performance and clinical implications of VI-RADS in detecting muscle invasion in bladder tumors: a prospective observational study. Indian J Urol.

[9] Lai AL, Law YM (2023). VI-RADS in bladder cancer: overview, pearls and pitfalls. Eur J Radiol.

[10] Naselli A, Hurle R, Paparella S (2018). Role of restaging transurethral resection for T1 non-muscle invasive bladder cancer: a systematic review and meta-analysis. Eur Urol Focus.

[11] van den Bosch S, Alfred Witjes J (2011). Long-term cancer-specific survival in patients with high-risk, non-muscle-invasive bladder cancer and tumour progression: a systematic review. Eur Urol.

[12] Panebianco V, Barchetti F, de Haas RJ, Pearson RA, Kennish SJ, Giannarini G, Catto JW (2016). Improving staging in bladder cancer: the increasing role of multiparametric magnetic resonance imaging. Eur Urol Focus.

[13] Wong VK, Ganeshan D, Jensen CT, Devine CE (2021). Imaging and management of bladder cancer. Cancers (Basel).

[14] Zhang X, Wang Y, Wang Y (2024). MRI evaluation of vesical imaging reporting and data system for bladder cancer after neoadjuvant chemotherapy. Cancer Imaging.

[15] Woo S, Suh CH, Kim SY, Cho JY, Kim SH (2017). Diagnostic performance of MRI for prediction of muscle-invasiveness of bladder cancer: a systematic review and meta-analysis. Eur J Radiol.

[16] Eusebi L, Masino F, Gifuni R, Fierro D, Bertolotto M, Cova MA, Guglielmi G (2023). Role of multiparametric-MRI in bladder cancer. Curr Radiol Rep.

[17] Ueno Y, Tamada T, Takeuchi M (2021). VI-RADS: multiinstitutional multireader diagnostic accuracy and interobserver agreement study. AJR Am J Roentgenol.

[18] Vaz A, Zaparolli M (2020). Diagnostic accuracy of retrospective application of the Vesical Imaging-Reporting and Data System: preliminary results. Radiol Bras.

[19] Del Giudice F, Vestri A, Fegatelli DA (2025). VI-RADS followed by photodynamic transurethral resection of non-muscle-invasive bladder cancer vs white-light conventional and second-resection: the 'CUT-less' randomised trial protocol. BJU Int.

[20] Wong-You-Cheong JJ, Woodward PJ, Manning MA, Sesterhenn IA (2006). Neoplasms of the urinary bladder: radiologic-pathologic correlation. Radiographics.

[21] Takeuchi M, Sasaki S, Ito M (2009). Urinary bladder cancer: diffusion-weighted MR imaging-accuracy for diagnosing T stage and estimating histologic grade. Radiology.

